# Association of tooth brushing behavior with risks of major chronic health outcomes: a scoping review

**DOI:** 10.1186/s12903-025-06332-4

**Published:** 2025-06-08

**Authors:** Hee-Jung Park, Nam-Hee Kim, Sun-Jung Shin, Hwa-Young Lee, Jin-Young Jeong

**Affiliations:** 1 Department of Dental Hygiene, College of Health Science, Kangwon National University, Samcheok, South Korea; 2 Department of Dental Hygiene, Yonsei University, Mirae Campus, Wonju, South Korea; 3 Department of Dental Hygiene, College of Dentistry, Gangneung Wonju National University, Gangneung, South Korea; 4 Graduate School of Public Health and Healthcare Management, The Catholic University of Korea, Seoul, South Korea; 5 Catholic Institute for Public Health and Healthcare Management, The Catholic University of Korea, Seoul, South Korea; 6 Hallym Research Institute of Clinical Epidemiology, Hallym University, Chuncheon, South Korea

**Keywords:** Tooth brushing, Oral hygiene behavior, Cardiovascular events, Stroke, Hypertension, Metabolic syndrome, Chronic kidney disease

## Abstract

**Background:**

Oral hygiene behavior has been increasingly recognized as a potential contributor to chronic disease prevention. This scoping review aimed to synthesize existing evidence on the associations between tooth brushing behavior (as a proxy for oral hygiene) and major chronic health outcomes, including cardiovascular events (e.g., myocardial infarction, atrial fibrillation, heart failure), stroke (ischemic, hemorrhagic, and subarachnoid), hypertension (HTN), metabolic syndrome (MetS), and chronic kidney disease (CKD).

**Methods:**

A comprehensive literature search was conducted using four databases: Ovid-MEDLINE, EMBASE, CINAHL, and the Cochrane Library. The study selection process followed the Preferred Reporting Items for Systematic Reviews and Meta-Analyses (PRISMA) 2020 guidelines. Two reviewers independently screened and selected eligible studies and extracted relevant data. A total of 142 full-text articles were assessed for eligibility.

**Results:**

Twenty-one studies were included in the final review. Most studies reported that a lower frequency of tooth brushing was associated with a higher risk of cardiovascular events, stroke, HTN, MetS, and CKD. Additionally, several studies suggested that maintaining good oral hygiene in individuals with HTN or diabetes mellitus (DM) may be associated with a reduced risk of cardiovascular complications.

**Conclusion:**

Frequent tooth brushing, as a key component of oral hygiene, may be associated with a reduced risk of several chronic health outcomes, particularly cardiovascular and metabolic diseases. However, the current body of evidence is predominantly based on observational studies. Further longitudinal and interventional research is warranted to clarify the directionality and potential causal pathways linking oral hygiene behavior to systemic health outcomes.

**Supplementary Information:**

The online version contains supplementary material available at 10.1186/s12903-025-06332-4.

## Background

Cardiovascular disease (CVD) remains the leading cause of death worldwide [1]. Currently, more than 70% of adults are affected by one or more forms of CVD, including hypertension (HTN), a major contributor to heart failure, coronary artery disease, and stroke [2]. In 2000, approximately 14 million deaths globally were attributed to CVD, a figure that increased to nearly 18 million by 2019 [3]. This upward trend is largely driven by increased life expectancy and the aging global population. In addition to the substantial health burden, CVD imposes considerable economic costs on individuals and healthcare systems [1].

The burden of CVD could be further alleviated through a deeper understanding of its association with oral health conditions [4–6]. Oral disease is a neglected, silent epidemic and a significant public health concern worldwide [7]. Integrating oral health into the broader context of general (systemic) health is strongly recommended [8]. Poor oral health, particularly periodontal disease, has been shown to increase the risk of CVD [9, 10], and these conditions share common risk factors, including smoking, diabetes mellitus (DM), and aging [11, 12].

Importantly, personal oral hygiene practices—particularly regular tooth brushing—are fundamental to preventing periodontal disease and may also influence cardiovascular health [11–13]. Mechanistically, inadequate tooth brushing facilitates the accumulation of dental biofilm, leading to periodontal inflammation and systemic immune responses, which may contribute to endothelial dysfunction and atherosclerosis.

A growing body of research has examined the association between infrequent tooth brushing and an elevated risk of CVD. For instance, a Scottish cohort study reported that frequent tooth brushing was associated with a lower risk of CVD events and reduced levels of inflammatory markers such as C-reactive protein [14]. Other cohort studies have found that individuals with neurodegenerative or cerebrovascular conditions who maintained regular oral hygiene experienced fewer cardiovascular events [15].

Furthermore, poor oral hygiene has also been linked to metabolic syndrome (MetS), a well-established risk factor for CVD. Kobayashi et al. (2012) reported that individuals who brushed their teeth only once daily had a higher risk of hypertriglyceridemia [16], while Matsui et al. (2017) observed a 40–50% reduction in MetS risk with increased brushing frequency [17]. Fujita et al. (2009) similarly found that infrequent tooth brushing was associated with a higher prevalence of MetS components, including DM, HTN, and dyslipidemia (DL) [18]. More recent large-scale cohort studies have also demonstrated associations between low tooth brushing frequency and increased risks of cardiovascular events, stroke, and chronic kidney disease (CKD) [19, 20].

Despite these findings, no scoping review has comprehensively synthesized the growing evidence linking tooth brushing behavior to systemic health outcomes. Therefore, the purpose of this review is to systematically examine existing research on the associations between tooth brushing behavior (as a proxy for oral hygiene) and major chronic health outcomes, including cardiovascular events (e.g., myocardial infarction, atrial fibrillation, heart failure), stroke (ischemic, hemorrhagic, and subarachnoid), HTN, MetS, and CKD.

The central research question addressed in this review is: *“What is the current state of knowledge regarding the impact of tooth brushing on cardiovascular and other chronic health outcomes?”* By addressing this question, the review aims to highlight the broader systemic implications of oral hygiene behavior and its potential role in chronic disease prevention.

## Methods

This scoping review was conducted in accordance with the Preferred Reporting Items for Systematic Reviews and Meta-Analyses (PRISMA) guidelines (2020 version) [21]. A comprehensive literature search was performed on June 30, 2024, covering all articles published up to and including that date.

The search strategy was applied to the literature databases Ovid-MEDLINE, Embase, Cochrane, CINAHL. Medical Subject Headings (MeSH) and EMTREE (the collection of standardized keywords used in Embase) terms were utilized to select the search descriptors. Boolean operators “AND” and “OR” were employed to refine the search strategy through various combinations. For example, the following search phrase was constructed for the CINAHL database: (“Oral hygiene” OR “Dental Devices, Home Care” OR “Toothbrushing”) AND (“Chronic Disease” OR “Heart Diseases” OR “Coronary Disease” OR “Cerebrovascular Disorders” OR “Stroke” OR “Diabetes Mellitus, Type 2” OR “Metabolic Syndrome” OR “Kidney Diseases”). A language filter was applied during the search process to include only studies published in English. No restrictions were placed on publication year. The detailed search terms used in this study can be found in Additional File 1.

Although HTN was not explicitly included in the database search strategy, relevant studies were captured through broader indexing categories such as CVD and chronic disease during the screening process. Therefore, studies addressing HTN -related outcomes were considered for inclusion based on their alignment with the research objectives.

After conducting the database searches, all potential articles were compiled in EndNote X11. Two reviewers, H.P. and N.K., independently screened the titles and abstracts for relevance. In addition to the database search, we performed a supplementary handsearch, including citation tracking of the references from included studies and consultation with experts in the field. This process yielded two additional studies [9, 15] that were not retrieved in the original database search but met the predefined inclusion criteria and were thus included in the final review. The decisions for inclusion or exclusion made by both reviewers were compared based on their review of the abstracts. Any discrepancies were discussed between the two reviewers until a consensus was reached on all items.

This scoping review was guided by a research question developed using the PCC (Population–Concept–Context) framework, as recommended by the Joanna Briggs Institute (JBI) methodology [22]. Specifically:

- Population (P): Adults aged 18 years and older.
- Concept (C): Tooth brushing behavior, as a proxy for oral hygiene.
- Context (C): Chronic health outcomes, including cardiovascular events (e.g., myocardial infarction, atrial fibrillation, heart failure), stroke (ischemic, hemorrhagic, and subarachnoid), hypertension, metabolic syndrome (MetS), and chronic kidney disease (CKD).

The framework was used to define the scope of the review and inform the inclusion and exclusion criteria. Studies were included if they met all the following criteria:

1. Reported empirical associations between tooth brushing behavior and systemic health outcomes;
2. Focused on cardiovascular disease (CVD) outcomes, stroke, hypertension (HTN), metabolic syndrome (MetS), or chronic kidney disease (CKD);
3. Conducted in adult populations (aged ≥ 18 years);
4. Applied observational or interventional study designs (e.g., cross-sectional, cohort, case-control, randomized trials);
5. Included clearly defined outcome measures and exposure assessments related to tooth brushing frequency or behavior.

Studies were excluded if they met any of the following criteria:

1. Focused exclusively on animal or in vitro models;
2. Did not present original empirical data (e.g., review articles, editorials, commentaries, protocols, or book chapters);
3. Evaluated tooth brushing only as a tool or technique, rather than as a behavioral exposure;
4. Reported outcomes outside the predefined scope (e.g., dental caries, periodontal indices, oral health-related quality of life, HbA1c levels);
5. Lacked methodological rigor, such as undefined brushing frequency categories, incomplete outcome definition, or unclear statistical analysis.

In this review, chronic systemic health outcomes were predefined and classified into five clinically relevant categories based on prior literature:

1. **CVD outcomes**: Myocardial infarction, atrial fibrillation, heart failure, and major adverse cardiovascular events (MACEs);
2. **Stroke**: Ischemic, hemorrhagic, or subarachnoid stroke subtypes;
3. **Hypertension (HTN)**: Defined by physician diagnosis, clinical blood pressure measures, or antihypertensive medication use;
4. **Metabolic Syndrome (MetS)**: Defined according to ATP III, AHA/NHLBI, or equivalent national guidelines (e.g., presence of ≥ 3 of the following: elevated waist circumference, high triglycerides, low HDL-C, high blood pressure, high fasting glucose);
5. **Chronic Kidney Disease (CKD)**: Diagnosed based on clinical coding (e.g., ICD codes) or reduced estimated glomerular filtration rate (eGFR < 60 mL/min/1.73 m²).

These standardized definitions were applied consistently across all stages of the review process, including study screening, data extraction, and synthesis.

Data were extracted using a standardized form in Microsoft Excel. Two reviewers independently extracted the following information from each study: author, publication year, country, study design, data source, study period, sample size, study population characteristics, participant age, exposure (predictors), tooth brushing frequency categories, outcome (criteria), controlled confounders, effect size, and summary of findings. Any discrepancies in data extraction were resolved through discussion.

## **Results**

**Fig. 1:**
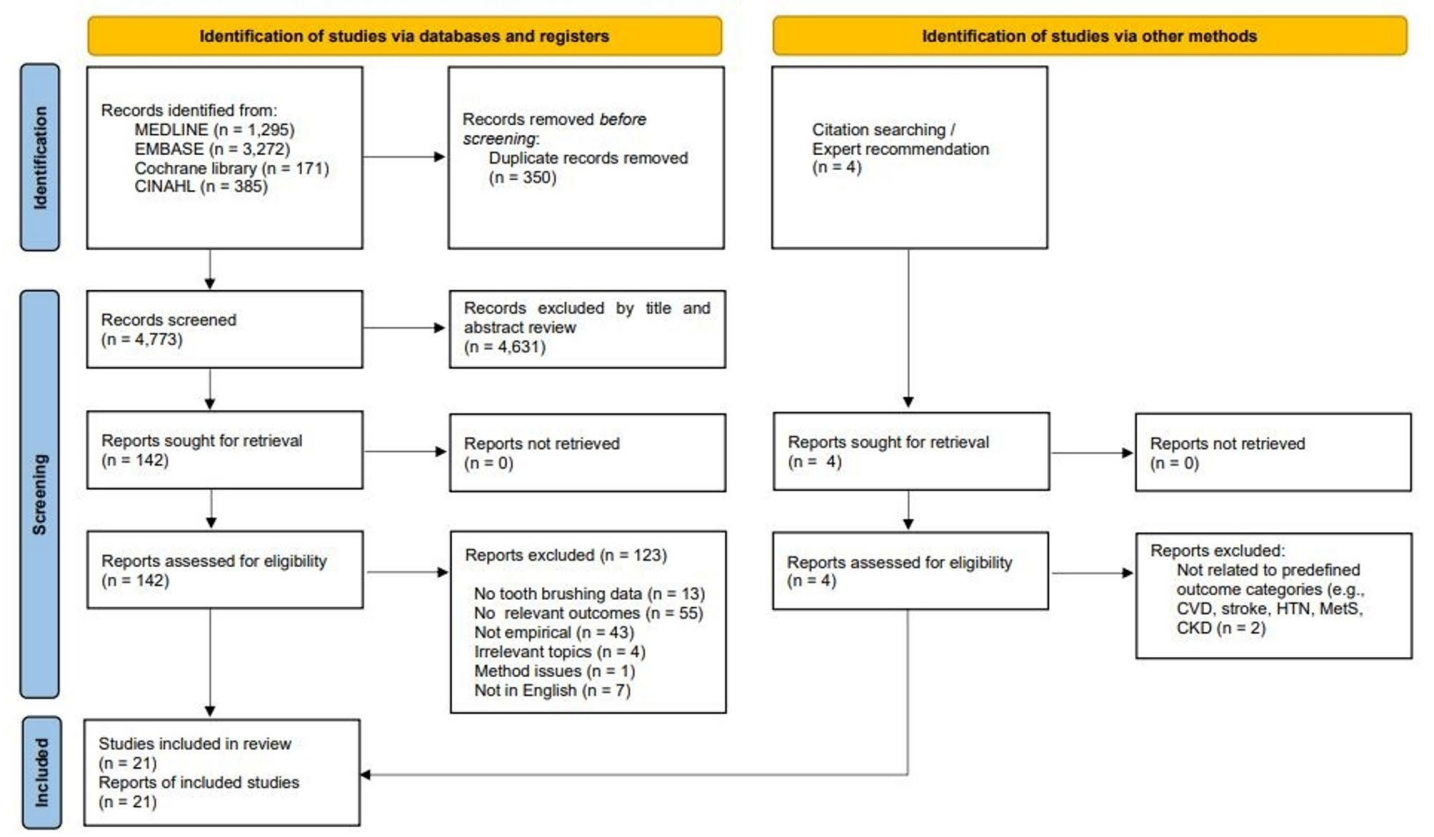
PRISMA 2020 flow diagram illustrating the study selection process for this scoping review. Records were identified through database searches (MEDLINE, EMBASE, Cochrane Library, and CINAHL) and additional handsearching (e.g., citation tracking and expert recommendations). Two studies were added through handsearching and included in the final review after full-text assessment

We initially identified 5,123 articles, of which 4,773 remained after duplicates were removed. During the screening of titles and abstracts, 4,631 articles were excluded, and 142 full-text articles were assessed for eligibility. Among them, 123 articles were excluded based on the inclusion and exclusion criteria. The detailed selection process is illustrated in Fig. 1.

The detailed selection process is illustrated in Fig. 1. In total, 21 studies were included—19 from database searches 2 through handsearching (e.g., citation tracking and expert recommendations). The characteristics of the included studies are summarized in Table 1.

**Table 1:** Basic characteristics of included studies (*n* = 21)

Author, Year	Country	Study design	Data source	Study period	Sample characteristics: sample size, sample descriptions	Participant age
Park et al., 2019 [11]	Korea	Prospective cohort	National Health Insurance System-National Health Screening Cohort	2002–2013	247,696	52 years
Matsui et al., 2022 [23]	Japan	Prospective cohort	Health screening examinations	2010–2016	692 Patients with CVD(*n* = 201)	63 ± 16 year
Chang et al., 2020 [24]	Korea	Prospective cohort	Korean National Health Insurance System-Health Screening Cohort	Median follow-up of 10.5 years	161,286	52.2 ± 8.7 years
Kim et al., 2022 [25]	Korea	Prospective cohort	Korean National Health Insurance Service-National Health Screening Cohort	2003–2015	52,677 Patients with hypertension	54.9 ± 9.5 years
Huh et al., 2023 [26]	Korea	Prospective cohort	Korean National Health Insurance Service-National Health Screening Cohort	2008–2017	173,927 Patients with diabetes	60.1 ± 10.7 years
Song et al., 2021 [27]	Korea	Prospective cohort	Nationwide health-screening program in South Korea	2012–2018	16,088 Patients with diabetes	59.3 ± 8.8 years
Kobayashi et al., 2020 [28]	Japan	Retrospective cohort study	Health check-ups	2005–2018	71,221	45.6 ± 12.2 years
de Oliveira et al., 2010 [14]	UK (Scotland)	Cross-sectional	Scottish Health Survey	1995, 1998, 2003	11,869	50 ± 11 years
Kajikawa et al., 2014 [29]	Japan	Cross-sectional	Health-screening examination	2012–2013	190	57 ± 18 years
Chang et al., 2021 [20]	Korea	Prospective cohort	Korean National Health Insurance data sharing service	2003–2015	206,602	53.5 ± 8.6 years
Zhuang et al., 2021 [15]	China	Prospective cohort	CKB cohort	2004–2008	512, 715	51.5 ± 10.6 years
Choi et al., 2015 [9]	Korea	Cross-sectional	Korea National Health and Nutrition Examination Survey	2008–2010	19,560	53.4 ± 12.2 years
Del Pinto et al., 2022 [30]	Italy	Cross-sectional	Italian Pharmacy Survey	2020	4,506	66.1 ± 37.8 years
Wang et al., 2022 [31]	China	Prospective cohort	The Guizhou Population Health Cohort	2010–2012	9,280	44.5 ± 15.2
Kuwabara et al., 2016 [32]	Japan	Cross-sectional	Health screening examinations	2004–2010	85,866	47.0 ± 11.5 years
Tanaka et al., 2018 [33]	Japan	Prospective cohort	Medical check-ups and dental examinations	5 years	3,722	44.5 ± 8.2 years
Kim et al., 2014 [34]	Korea	Cross-sectional	Korea National Health and Nutrition Examination Survey	2008–2010	18,742	MetS(+): 40.9 ± 0.2 years MetS(-): 53.7 ± 0.3 years
Kobayashi et al., 2012 [16]	Japan	Cross-sectional and longitudinal study	Not specified directly, but involved a cohort from a health examination program	Cross-sectional analysis at baseline, with a longitudinal follow-up of 3 years	Cross-sectional: 925 Longitudinal: 685	Frequency of daily tooth brushing ≤1/day (45.0 years), 2/day (43.0 years), ≥ 3/day (43.0 years)
Tsutsumi et al., 2015 [35]	Japan	Cross-sectional	Kurume medical check-up DB	Not specified, analysis of medical check-up records from a large database	12,548	46.3 ± 7.5 years
Esfanjani et al., 2023 [36]	Iran	Cross-sectional	Azar Cohort	2014–2017	15,006	MetS(+): 52.3 ± 9 years MetS(-): 48.3 ± 9.1 years
Chang et al., 2021 [19]	Korea	Prospective cohort	Korean National Health Insurance data sharing service	2003–2015	158,495	52.3 ± 8.8 years

The included studies were published between 2010 and 2023 and primarily originated from Asian populations, including nine studies from Korea, seven from Japan, two from China, and one from Iran. Two additional studies were conducted in Europe (Scotland and Italy). Sample sizes ranged from 190 to 247,696 participants. Most studies involved adult participants across a broad age range, although a few categorized age based on oral hygiene behavior or the presence of health conditions [16, 26, 34, 36].

Eight studies employed cross-sectional designs [9, 14, 29, 30, 32, 34–36], while one study combined cross-sectional and longitudinal analyses [16]. Most studies were based on large-scale national surveys or cohort data [9, 11, 15, 19, 20, 24–28, 30–36]. Three studies specifically targeted high-risk populations, such as individuals with hypertension or diabetes mellitus [25–27].

All studies assessed tooth brushing frequency as the primary indicator of oral hygiene behavior. Most used predefined categories (e.g., once, twice, or ≥ 3 times per day), with some including additional options such as brushing after every meal [9, 28, 32].

To facilitate structured synthesis, we categorized the included studies into five predefined outcome domains:

1. **Cardiovascular Events (9 studies)**: Most studies reported that higher tooth brushing frequency was associated with a lower risk of cardiovascular outcomes such as myocardial infarction, atrial fibrillation, and heart failure. These associations were particularly evident in large-scale cohort studies [11, 14, 23–29].
2. **Stroke (2 studies)**: Two studies identified significant associations between frequent tooth brushing and a lower risk of stroke, including ischemic and hemorrhagic types [15, 21].
3. **Hypertension (3 studies)**: Three studies demonstrated that infrequent tooth brushing was associated with a higher prevalence or incidence of hypertension [9, 30, 31].
4. **Metabolic Syndrome (6 studies)**: Several studies consistently reported that low tooth brushing frequency was associated with increased risk of MetS, based on ATP III or AHA/NHLBI definitions [16, 32–36].
5. **Chronic Kidney Disease (1 study)**: One study found that brushing ≥ 3 times per day was associated with reduced CKD risk, although two others studies reported null findings, indicating inconsistency in this outcome domain [20].

The detailed results categorized by outcome are presented in Table 2 and Table 3.

**Table 2:** Summary of predictors, outcome criteria, and confounders by outcome category (*n* = 21)

Authors, Year	Outcome category	Predictors (Tooth brushing behavior)	Tooth brushing frequency	Outcome criteria	Controlled confounders
Park et al., 2019 [11]	Cardiovascular events	Tooth brushing frequency	0–1/day versus 2/day versus ≥ 3/day	Composite major cardiovascular events - Cardiac death, - Acute myocardial - Infarction, - Stroke - Heart failure	Age, sex, income, exercise, BMI, systolic BP, HTN, DM, DL, renal disease, malignancy, current smoking, oral health status (periodontal disease, caries, tooth loss)
Matsui et al., 2022 [23]	Cardiovascular events	Tooth brushing frequency & duration	< twice/day and < 2 min/procedure < twice/day or < 2 min/procedure ≥twice/day and ≥ 2 min/procedure	Major Adverse Cardiovascular Events (MACEs): - Cardiovascular death - Non-fatal myocardial infarction - Heart failure hospitalization - Stroke	Age (> 65), sex, BMI, hypertension, DM, smoking history, pre-existing CVD (CAD, HF, stroke)
Chang et al., 2020 [24]	Cardiovascular events	Tooth brushing frequency	0–1/day versus 2/day versus ≥ 3/day	Incident atrial fibrillation Incident heart failure	Age, sex, socioeconomic status, regular exercise, alcohol consumption, BMI, SBP, diabetes, dyslipidemia, smoking, renal disease, history of cancer, fasting glucose, total cholesterol, liver enzymes, proteinuria, periodontal disease, tooth brushing, dental visit, professional cleaning, number of missing teeth
Kim et al., 2022 [25]	Cardiovascular events	Tooth brushing frequency	0–1/day versus ≥ 2/day	Composite of stroke - Ischemic - Hemorrhagic - Myocardial infarction	Age, sex, household income, smoking, alcohol, exercise, BMI, SBP, total cholesterol, diabetes, atrial fibrillation, renal disease, malignancy
Huh et al., 2023 [26]	Cardiovascular events	Tooth brushing frequency	0–1/day versus ≥ 2/day	Incident heart failure	Age, sex, smoking, alcohol, physical activity, income, BMI, dyslipidemia, CKD, CVD, number of oral antidiabetic medications, insulin use
Song et al., 2021 [27]	Cardiovascular events	Tooth brushing frequency	0–1/day versus ≥ 2/day	Cardiovascular events - Cerebral infarction - Myocardial infarction	Gender, age, household income, BMI, smoking status, alcohol, physical activity, HTN, fasting glucose, total cholesterol
Kobayashi et al., 2020 [28]	Cardiovascular events	Tooth brushing frequency	0/day versus 1/day versus 1–2/day versus every time after a meal	Composite cardiovascular events - Acute coronary syndrome + Stroke - Stroke; acute coronary syndrome	Age, sex, smoking, alcohol, exercise, BMI, hypertension, diabetes, dyslipidemia
de Oliveira et al., 2010 [14]	Cardiovascular events (and inflammatory markers as mechanism)	Tooth brushing frequency	< 1/day versus 1/day versus 2/day	Composite cardiovascular events - fatal + non-fatal CVD (CHD, stroke, heart failure, revascularization) - Inflammatory markers: high-sensitivity C-reactive protein and fibrinogen	Age, sex, socioeconomic status, smoking, physical activity, dental visits, BMI, family history of CVD, hypertension, diabetes, acute infections
Kajikawa et al., 2014 [29]	Cardiovascular events	Tooth brushing frequency	< 1/day versus ≥ 2/day	Endothelial dysfunction	Age, sex, diabetes mellitus, dyslipidemia
Chang et al., 2021 [20]	Stroke	Tooth brushing frequency	0–1/day versus 2/day versus ≥ 3/day	Stroke occurrence - Cerebral infarction - Cerebral hemorrhage - Subarachnoid hemorrhage	Age, sex, income level, exercise, alcohol use, smoking, BMI, HTN, DM, dyslipidemia, renal disease, malignancy, SBP, cholesterol, FBG, AST/ALT, GGT, proteinuria, oral hygiene indicators
Zhuang et al., 2021 [15]	Stroke	Tooth brushing frequency	< 1 time/day versus ≥ 2	Stroke occurrence - Intra cerebral hemorrhage- - Subarachnoid hemorrhage	Age, sex, region, education, income, marital status, smoking, alcohol intake, physical activity (MET), fruit/vegetable/meat intake, BMI, waist-hip ratio, systolic BP
Choi et al., 2015 [9]	Hypertension	Tooth brushing frequency	≤ 1/day versus after every meal	Hypertension prevalence Hypertension control	Age, sex, BMI, smoking, drinking, physical activity, education, income, and presence of periodontitis
Del Pinto et al., 2022 [30]	Hypertension	Tooth brushing frequency	1/day versus 2/day versus > 3/day versus ≥ 3/day	Prevalent hypertension Uncontrolled hypertension	Age, sex, BMI, smoking, diabetes, hypercholesterolemia, physical activity, daily fruit/vegetable intake, toothbrush type
Wang et al., 2022 [31]	Hypertension	Tooth brushing frequency	0/day versus < 1/day versus ≥ 1/day	Incident hypertension	Age, sex, BMI, smoking, exercise
Kuwabara et al., 2016 [32]	Metabolic Syndrome	Tooth brushing frequency	1/day or rarely versus < 1/day versus every time after a meal	Diabetes mellitus Dyslipidemia	Age, gender, BMI, lifestyle habits (smoking, drinking, walk time, sleep time), comorbidities (HT, DM, DL, HUA, CKD)
Tanaka et al., 2018 [33]	Metabolic syndrome	Tooth brushing frequency	≤ 1/day versus 2/day versus > 3/day	Metabolic syndrome	Age, gender, periodontal status, number of present teeth, occupational status, smoking, alcohol consumption, physical activity, dental check-up regularity, dietary behavior, salty food preference, baseline MetS components
Kim et al., 2014 [34]	Metabolic syndrome	Tooth brushing frequency	≤ 1/day versus 2/day versus ≥ 3/day	Metabolic syndrome	Age, sex, income, education, smoking, alcohol intake, physical activity, plus individual MetS components in fully adjusted models
Kobayashi et al., 2012 [16]	Metabolic syndrome	Tooth brushing frequency	≤ 1/day versus 2/day ≥ 2/day versus ≥ 3/day	Metabolic syndrome	Age, sex, smoking, alcohol intake, physical activity, occupation, education level, depressive symptoms, breakfast eating, total caloric intake, mutual adjustment of MetS components
Tsutsumi et al., 2015 [35]	Metabolic syndrome	Tooth brushing frequency	Male: 0 versus 1/day versus 2/day versus > 2 /day Female: 1/day versus 2/day versus > 2 /day	Metabolic syndrome	Age, exercise during holidays, favorite seasoning, soup consumption, sugar in coffee, interest in weight loss, housekeeping during holidays, worries about job, eating speed, dinner preparation, problem-solving behavior
Esfanjani et al., 2023 [36]	Metabolic syndrome	Tooth brushing behavior	0/day versus 1/day versus 2/day versus 3/day	Metabolic syndrome	Age, gender, education level, wealth score index
Chang et al., 2021 [19]	Chronic kidney disease	Tooth brushing frequency	0–1/day versus 2/day versus ≥ 3/day	Incident chronic kidney disease	Age, sex, income level, smoking, alcohol, physical activity, BMI, HTN, DM, DL, malignancy, atrial fibrillation, heart failure, SBP, FBG, total cholesterol, AST, ALT, GGT

**Table 3:** Effect sizes and main findings of included studies according to outcome category

Authors, Year	Outcome category	Effect size	Summary of findings
Park et al., 2019 [11]	Cardiovascular events	- ≥3/day: HR 0.81 (95% CI: 0.78–0.84) **-** 2/day: HR 0.91 (95% CI: 0.88–0.94)	Frequent tooth brushing was significantly associated with reduced cardiovascular risk.
Matsui et al., 2022 [23]	Cardiovascular events	**-** MACEs (Low frequency & short duration): HR 3.06 (95% CI: 1.24–7.63) - Death from CVD: HR 7.55 (95% CI: 1.23–62.4) **-** Heart failure hospitalization HR 3.81 (95% CI: 1.06–14.4) **-** <1.5 min/day: HR 2.98 (95% CI: 1.33–6.38)	Combination of decreased frequency and short duration of tooth brushing is independently associated with increased MACEs risk. Higher brushing duration is protective.
Chang et al., 2020 [24]	Cardiovascular events	- ≥3 /day: Atrial fibrillation HR 0.90 (95% CI: 0.83–0.98) - ≥3 /day: Heart failure HR 0.88 (95% CI: 0.83–0.94)	Frequent tooth brushing (≥ 3/day) was associated with reduced risk of atrial fibrillation and heart failure.
Kim et al., 2022 [25]	Cardiovascular events	- ≥2 /day: Composite CVD HR 0.88 (95% CI: 0.81–0.96) - ≥2 /day: Stroke HR 0.87 (95% CI: 0.79–0.96) - ≥2 /day: Hemorrhagic stroke HR 0.77 (95% CI: 0.64–0.92) - ≥2 /day Ischemic stroke HR 0.91 (95% CI: 0.82–1.03)	Frequent brushing associated with lower composite cardiovascular outcomes.
Huh et al., 2023 [26]	Cardiovascular events	**-** ≥2/day: Heart failure HR 0.90 (95% CI: 0.82–0.98) **-** Combined good oral hygiene care (cleaning + brushing): HR 0.85 (95% CI: 0.75–0.95)	Among diabetes patients, regular brushing and professional cleaning reduce heart failure risk, even in presence of dental disease.
Song et al., 2021 [27]	Cardiovascular events	- ≥2/day: Myocardial infarction HR 0.79 (95% CI: 0.70–0.90) - ≥2/day: Cerebral infarction: HR 0.81 (95% CI: 0.69–0.94)	Among diabetic patients, frequent tooth brushing reduced the risk of stroke and myocardial infarction. Association more pronounced for stroke than myocardial infarction.
Kobayashi et al., 2020 [28]	Cardiovascular events	− 1/day: OR 1.11 (95% CI: 0.98–1.26) - 1–2/day: OR 1.03 (95% CI: 0.92–1.15)	Less frequent tooth brushing was associated with a higher risk of cardiovascular events in a dose-dependent manner. Stroke risk remained significant after full adjustment.
de Oliveira et al., 2010 [14]	Cardiovascular events	− 1/day: Cardiovascular events HR 1.3 (95% CI: 1.0–1.5) - <1/day: Cardiovascular events HR 1.7 (95% CI: 1.3–2.3) - <1/day: CRP β = 0.04 (95% CI: 0.01–0.08) - <1/day: Fibrinogen β = 0.08 (95% CI: -0.01–0.18)	Less frequent tooth brushing is associated with increased risk of cardiovascular events and elevated inflammatory markers (high-sensitivity C-reactive protein, fibrinogen).
Kajikawa et al., 2014 [29]	Cardiovascular events	- <1/day: flow-mediated vasodilation 3.3 ± 2.2% - ≥2/day: flow-mediated vasodilation 5.0 ± 3.0%	Decreased frequency of tooth brushing is independently associated with endothelial dysfunction. flow-mediated vasodilation was significantly lower in the low brushing group.
Chang et al., 2021 [20]	Stroke	≥ 3/day: HR 0.78 (95% CI: 0.73–0.84)	Frequent tooth brushing (≥ 3/day) significantly reduced total stroke risk (especially cerebral infarction).
Zhuang et al., 2021 [15]	Stroke	- <1/day: Stroke HR 1.08 (95% CI: 1.05–1.12) - <1/day: Intracerebral hemorrhage HR 1.18 (95% CI: 1.11–1.26) - <1/day: Subarachnoid hemorrhage HR 0.97 (95% CI: 0.74–1.26)	Infrequent tooth brushing (< 1/day) was associated with an increased risk of intracerebral hemorrhage. Findings suggest poor oral hygiene behavior may increase the risk of stroke.
Choi et al., 2015 [9]	Hypertension	≤ 1/day: OR 1.20 (95% CI: 1.03–1.38)	Poor oral hygiene behavior is independently associated with higher prevalence of hypertension. The effect is observed even in individuals without periodontitis, suggesting a possible role of oral hygiene in hypertension prevention and control.
Del Pinto et al., 2022 [30]	Hypertension	- ≥3/day: OR 0.81 (95% CI: 0.70–0.94) - Manual brushing ≥ 3/day: OR 0.83 (95% CI: 0.71–0.98)	Frequent tooth brushing (≥ 3/day) is independently associated with lower BP and reduced odds of prevalent/uncontrolled hypertension. Best effect observed in electric brushing ≥ 3/day group.
Wang et al., 2022 [31]	Hypertension	− 1/day: HR 0.77 (95% CI: 0.60–0.98) - ≥2/day: HR 0.55 (95% CI: 0.42–0.73)	Brushing teeth at least twice a day was associated with significantly lower risk of incident hypertension in a large-scale 10-year prospective cohort study in Southwest China. Dose-response association observed.
Kuwabara et al., 2016 [32]	Metabolic syndrome	**− 1**/day: Diabetes mellitus OR 2.03 (95% CI: 1.29–3.21) - 1/day: Dyslipidemia OR 1.50 (95% CI: 1.06–2.14)	Less frequent tooth brushing was significantly associated with higher prevalence of diabetes mellitus and dyslipidemia.
Tanaka et al., 2018 [33]	Metabolic syndrome	- ≥3/day: OR 0.64 (95% CI: 0.45–0.92) - 2/day: OR 0.83 (95% CI: 0.65–1.05)	Higher toothbrushing frequency is significantly associated with reduced risk of developing metabolic syndrome over a 5-year follow-up period.
Kim et al., 2014 [34]	Metabolic syndrome	- ≤1/day: Metabolic syndrome OR 1.23 (95% CI: 1.04–1.47) - ≤1/day: Abdominal obesity OR 1.38 (95% CI: 1.25–1.52) - ≤1/day: Hyperglycemia OR 1.14 (95% CI: 1.04–1.25)	Infrequent tooth brushing (≤ 1/day) was significantly associated with increased risk of metabolic syndrome and its components (abdominal obesity, hyperglycemia, and high blood pressure). Strong dose–response relationship noted.
Kobayashi et al., 2012 [16]	Metabolic syndrome	**Cross-sectional analysis** - ≥3/day: OR 0.47 (95% CI: 0.24–0.92) - 2/day: OR 0.71 (95% CI: 0.48–1.05) **Longitudinal analysis** - ≥3/day: OR 0.43 (95% CI: 0.19–0.97) - 2/day: OR 0.80 (95% CI: 0.49–1.31)	Higher frequency of tooth brushing was associated with lower risk of both prevalent and incident metabolic syndrome. Among metabolic syndrome components, triglyceride level showed the most consistent inverse association.
Tsutsumi et al., 2015 [35]	Metabolic syndrome	**Males** - 1/day: OR 0.57 (95% CI: 0.40–0.81) - 2/day: OR 0.50 (95% CI: 0.35–0.71) - >2 /day: OR 0.42 (95% CI: 0.29–0.61) **Females** - 2/day: OR 0.65 (95% CI: 0.48–0.87) - >2 /day: OR 0.44 (95% CI: 0.32–0.62)	Tooth brushing frequency was inversely associated with metabolic syndrome risk in both men and women, demonstrating a clear dose–response relationship. Statistical significance was retained after adjustment for extensive lifestyle-related confounders.
Esfanjani et al., 2023 [36]	Metabolic syndrome	− 0/day: Metabolic syndrome OR 1.18 (95% CI: 1.05–1.33) - 0/day: Abdominal obesity OR 1.28 (95% CI: 1.13–1.44) - 0/day: Hyperglycemia OR 1.30 (95% CI: 1.16–1.47) - Irregular brushing: Metabolic syndrome OR 1.23 (95% CI: 1.10–1.37) - Irregular brushing: Abdominal obesity OR 1.38 (95% CI: 1.23–1.54) - Irregular brushing: Hypertriglyceridemia OR 1.18 (95% CI: 1.07–1.31) - Irregular brushing: Hyperglycemia OR 1.19 (95% CI: 1.06–1.33)	Infrequent tooth brushing was significantly associated with increased odds of metabolic syndrome and its components (obesity, hyperglycemia, hypertriglyceridemia). Brushing appears to reduce systemic inflammatory risk pathways contributing to metabolic syndrome.
Chang et al., 2021 [19]	Chronic kidney disease	- ≥3/day: HR 0.90 (95% CI: 0.83–0.99) - 2/day: HR 0.91 (95% CI: 0.83–1.01)	Increased frequency of tooth brushing was significantly associated with reduced chronic kidney disease risk in a large-scale Korean cohort. No significant interaction effects by comorbidities.

HR, hazard ratio; CI, confidence interval; OR, odds ratio

## Discussion

In this scoping review, we synthesized available studies on the association between tooth brushing frequency and cardiovascular disease (CVD) outcomes, as well as related chronic conditions including metabolic syndrome (MetS), stroke, hypertension (HTN), and chronic kidney disease (CKD). A total of 21 studies were included in our analysis. Most studies indicated that infrequent tooth brushing is associated with elevated risks of myocardial infarction, stroke (ischemic, hemorrhagic, and subarachnoid), atrial fibrillation, heart failure, MetS, HTN, and CKD.

MetS, characterized by high blood pressure and abnormal lipid profiles, is a recognized risk factor for CVD [16, 34]. Several studies in this review found a relationship between low frequency of tooth brushing and higher risk of MetS [16, 32–36]. This may be explained by the biological pathway whereby inadequate oral hygiene facilitates plaque accumulation, which leads to gingival inflammation and systemic inflammatory responses that influence insulin resistance and lipid metabolism. Periodontal pathogens such as *Porphyromonas gingivalis* stimulate pro-inflammatory cytokines and endotoxins, contributing to immune dysregulation and metabolic dysfunction [10]. Furthermore, previous clinical trials have demonstrated that increased tooth brushing frequency improves gingival health indicators, including bleeding on probing and plaque indices, supporting the relevance of tooth brushing frequency as a behavioral marker of actual oral hygiene status [37, 38].

In particular, individuals with diabetes mellitus (DM) may experience stronger protective effects from regular tooth brushing. This may be explained by immunometabolic pathways in which poor oral hygiene exacerbates systemic inflammation, worsening glycemic control and increasing CVD risk. Inflammatory mediators from periodontal disease have been shown to worsen insulin resistance and promote endothelial dysfunction, both of which are involved in cardiovascular pathogenesis [26, 27, 39].

It is important to note that improving oral hygiene in patients with HTN and DM could potentially lower the risk of cardiovascular events, such as stroke and myocardial infarction [25–27]. This correlation may be due to the fact that conditions like DM and HTN, along with their complications, can affect adherence to health behaviors, including physical activity, dietary control, and regular tooth brushing [40]. Previous research suggests that the severity and susceptibility of disease may increase the need for diligent health management practices [41, 42]. Furthermore, this association may be partly explained by the fact that individuals who brush their teeth regularly tend to be more health-conscious, which in turn contributes to a lower cardiovascular risk profile. One study included in this review suggested that people with heightened health awareness tend to practice frequent tooth brushing and adopt other healthy behaviors beneficial for cardiovascular health [28].

A consistent association was also found between tooth brushing frequency and reduced risk of cardiovascular events, including myocardial infarction, atrial fibrillation, and heart failure [11, 14, 24–28]. These findings were particularly supported by longitudinal cohort studies involving large population-based samples. Regarding stroke, both ischemic and hemorrhagic subtypes were found to be inversely associated with tooth brushing frequency in multiple studies [15, 20], further supporting the potential role of oral hygiene behavior in cerebrovascular disease prevention.

With respect to CKD, the evidence was less consistent. While one large-scale study reported a significant protective association between frequent brushing and lower CKD risk, other studies found null associations [19]. This highlights the need for further interventional research to clarify potential causal mechanisms between oral hygiene and kidney function.

It is also important to consider alternative explanations for the observed associations. Tooth brushing frequency may reflect general health consciousness, health literacy, or other positive health behaviors rather than a direct biological effect. Individuals who engage in regular tooth brushing may also adhere to other preventive health behaviors, such as maintaining a healthy diet, engaging in physical activity, and participating in routine medical checkups. These shared behavioral determinants should be considered in future studies to disentangle independent effects of oral hygiene from confounding lifestyle factors [43, 44].

Taken together, our findings highlight the potentially important role of tooth brushing and oral hygiene behavior in chronic disease prevention. Future studies should explore these relationships using rigorous longitudinal and interventional designs to confirm causality and inform integrated strategies for systemic health promotion through oral hygiene improvement.

Some strengths and limitation of our current work are worth noting. This is the first scoping review to examine the associations between tooth brushing and specific CVD outcomes and related chronic conditions. However, a meta-analysis was not feasible due to substantial methodological and clinical heterogeneity across the included studies. Specifically, the studies differed in terms of outcome definitions (e.g., ardiovascular events, stroke, MetS, HTN, CKD), exposure measurements (e.g., frequency of tooth brushing categorized as once, twice, or three or more times per day; brushing after every meal), population characteristics (e.g., general adult populations versus individuals with hypertension or diabetes), and study designs (cross-sectional versus longitudinal). Furthermore, the effect sizes were reported in various statistical formats such as odds ratios (OR) and hazard ratios (HR) with different sets of confounders controlled in multivariable models. These variations limited the comparability of findings and precluded a quantitative synthesis using meta-analysis. Therefore, a narrative synthesis was employed to summarize and interpret the existing evidence. Additionally, this review may have introduced language bias by including only studies published in English, potentially excluding relevant studies in other languages.

Although some previous studies have investigated the preventive role of tooth brushing in relation to poor oral health and CVD risk, our review specifically focuses on the association between tooth brushing, CVD, and related chronic conditions using a structured outcome-based synthesis. Despite these limitations, our review provides a comprehensive overview of the current literature and highlights the potential systemic health benefits of oral hygiene behaviors.

## Conclusions

This scoping review highlights that frequent tooth brushing, as an essential component of oral hygiene behavior, is associated with reduced risks of several chronic health outcomes, including cardiovascular events (e.g., myocardial infarction, atrial fibrillation, and heart failure), stroke, hypertension (HTN), metabolic syndrome (MetS), and chronic kidney disease (CKD). These findings suggest that oral hygiene behavior, particularly frequent tooth brushing, may play a preventive role in systemic health. However, the associations identified in this review are predominantly based on observational studies and cannot establish causality. Further longitudinal and interventional research is warranted to clarify the temporal relationships, elucidate underlying biological mechanisms, and confirm causal pathways linking oral hygiene behavior to systemic health.

## Electronic supplementary material

Below is the link to the electronic supplementary material.

Supplementary Material 1

## Data Availability

No datasets were generated or analysed during the current study.

## Abbreviations

CKD: Chronic kidney disease
CVD: Cardiovascular disease
DM: Diabetes mellitus
DL: Dyslipidemia
HDL-C: High-density lipoprotein cholesterol
HR: Hazard ratio
HTN: Hypertension
MACEs: Major adverse cardiovascular events
MeSH: Medical Subject Headings
MetS: Metabolic syndrome
OR: Odds ratio
PRISMA: Preferred Reporting Items for Systematic Reviews and Meta-Analyses
eGFR: Estimated glomerular filtration rate

## References

[1] Townsend N, Kazakiewicz D, Lucy Wright F, Timmis A, Huculeci R, Torbica A, et al. Epidemiology of cardiovascular disease in Europe. Nat Rev Cardiol. 2022;19(2):133–43. 10.1038/s41569-021-00607-3. 34497402 10.1038/s41569-021-00607-3

[2] Tran DMT, Lekhak N, Gutierrez K, Moonie S. Risk factors associated with cardiovascular disease among adult Nevadans. PLoS ONE. 2021;16(2):e0247105. 10.1371/journal.pone.0247105. 33596242 10.1371/journal.pone.0247105 PMC7888645

[3] Roth GA, Mensah GA, Johnson CO, Addolorato G, Ammirati E, Baddour LM, et al. Global burden of cardiovascular diseases and risk factors, 1990–2019: update from the GBD 2019 study. J Am Coll Cardiol. 2020;76(25):2982–3021. 10.1016/j.jacc.2020.11.010. 33309175 10.1016/j.jacc.2020.11.010 PMC7755038

[4] Genco R, Offenbacher S, Beck J. Periodontal disease and cardiovascular disease: epidemiology and possible mechanisms. J Am Dent Assoc. 2002;133. 10.14219/jada.archive.2002.0375.:14S-22S. 10.14219/jada.archive.2002.0375 12085720

[5] Jansson L, Lavstedt S, Frithiof L, Theobald H. Relationship between oral health and mortality in cardiovascular diseases. J Clin Periodontol. 2001;28(8):762–8. 10.1034/j.1600-051x.2001.280807.x. 11442736 10.1034/j.1600-051x.2001.280807.x

[6] Dietrich T, Webb I, Stenhouse L, Pattni A, Ready D, Wanyonyi KL, et al. Evidence summary: the relationship between oral and cardiovascular disease. Br Dent J. 2017;222(5):381–85. 10.1038/sj.bdj.2017.224. 28281612 10.1038/sj.bdj.2017.224

[7] Dye BA. The global burden of oral disease: research and public health significance. J Dent Res. 2017;96(4):361–63. 10.1177/0022034517693567. 28318392 10.1177/0022034517693567 PMC6728669

[8] Seitz MW, Listl S, Bartols A, Schubert I, Blaschke K, Haux C, et al. Current knowledge on correlations between highly prevalent dental conditions and chronic diseases: an umbrella review. Prev Chronic Dis. 2019;16:E132. 10.5888/pcd16.180641. 31560644 10.5888/pcd16.180641 PMC6795069

[9] Choi HM, Han K, Park YG, Park JB. Associations among oral hygiene behavior and hypertension prevalence and control: the 2008 to 2010 Korea National health and nutrition examination survey. J Periodontol. 2015;86(7):866–73. 10.1902/jop.2015.150025. 25741579 10.1902/jop.2015.150025

[10] Sanz M, Marco del Castillo A, Jepsen S, Gonzalez-Juanatey JR, D’Aiuto F, Bouchard P, et al. Periodontitis and cardiovascular diseases: consensus report. J Clin Periodontol. 2020;47(3):268–88. 10.1111/jcpe.13189. 32011025 10.1111/jcpe.13189 PMC7027895

[11] Park SY, Kim SH, Kang SH, Yoon CH, Lee HJ, Yun PY, et al. Improved oral hygiene care attenuates the cardiovascular risk of oral health disease: a population-based study from Korea. Eur Heart J. 2019;40(14):1138–45. 10.1093/eurheartj/ehy836. 30561631 10.1093/eurheartj/ehy836

[12] Frisbee SJ, Chambers CB, Frisbee JC, Goodwill AG, Crout RJ. Association between dental hygiene, cardiovascular disease risk factors and systemic inflammation in rural adults. J Dent Hyg. 2010;84(4):177–84. 21047463

[13] Lee SK, Hwang SY. Oral health in adults with coronary artery disease and its risk factors: a comparative study using the Korea National health and nutrition examination survey data. BMC Cardiovasc Disord. 2021;21(1):1–11. 10.1186/s12872-021-01878-x. 33541275 10.1186/s12872-021-01878-x PMC7863432

[14] de Oliveira C, Watt R, Hamer M. Toothbrushing, inflammation, and risk of cardiovascular disease: results from Scottish health survey. BMJ. 2010;340:c2451. 10.1136/bmj.c2451. 20508025 10.1136/bmj.c2451 PMC2877809

[15] Zhuang Z, Gao M, Lv J, Yu C, Guo Y, Bian Z, et al. Associations of toothbrushing behaviour with risks of vascular and nonvascular diseases in Chinese adults. Eur J Clin Invest. 2021;51(12):e13634. 10.1111/eci.13634. 34152010 10.1111/eci.13634 PMC7616900

[16] Kobayashi Y, Niu K, Guan L, Momma H, Guo H, Cui Y, et al. Oral health behavior and metabolic syndrome and its components in adults. J Dent Res. 2012;91(5):479–84. 10.1177/0022034512440707. 22378694 10.1177/0022034512440707

[17] Matsui S, Kajikawa M, Maruhashi T, Iwamoto Y, Iwamoto A, Oda N, et al. Decreased frequency and duration of tooth brushing is a risk factor for endothelial dysfunction. Int J Cardiol. 2017;241:30–4. 10.1016/j.ijcard.2017.03.049. 28341376 10.1016/j.ijcard.2017.03.049

[18] Fujita M, Ueno K, Hata A. Lower frequency of daily teeth brushing is related to high prevalence of cardiovascular risk factors. Exp Biol Med. 2009;234(4):387–94. 10.3181/0809-RM-265. 10.3181/0809-RM-265 19176866

[19] Chang Y, Lee JS, Woo HG, Ryu DR, Kim JW, Song TJ. Improved oral hygiene care and chronic kidney disease occurrence: A nationwide population-based retrospective cohort study. Medicine. 2021;100(47):e27845. 10.1097/MD.0000000000027845. 34964752 10.1097/MD.0000000000027845 PMC8615368

[20] Chang Y, Woo HG, Lee JS, Song TJ. Better oral hygiene is associated with lower risk of stroke. J Periodontol. 2021;92(1):87–94. 10.1002/JPER.20-0053. 32432793 10.1002/JPER.20-0053

[21] Page MJ, McKenzie JE, Bossuyt PM, Boutron I, Hoffmann TC, Mulrow CD et al. The PRISMA 2020 statement: an updated guideline for reporting systematic reviews. BMJ. 2021;372:n71. 10.1136/bmj.n71. 10.1136/bmj.n71 PMC8005924 33782057

[22] Peters MD, Marine C, Tricco AC, Pollock D, Munn Z, Alexander L, et al. Updated methodological guidance for the conduct of scoping reviews. JBI Evid Synth. 2020;18(10):2119–26. 10.11124/JBIES-20-00167. 33038124 10.11124/JBIES-20-00167

[23] Matsui S, Maruhashi T, Kishimoto S, Kajikawa M, Mohamad Yusoff F, Nakashima A, et al. Poor tooth brushing behavior is associated with high risk of cardiovascular events: a prospective observational study. Int J Cardiol. 2022;350:111–7. 10.1016/j.ijcard.2021.12.056. 34979151 10.1016/j.ijcard.2021.12.056

[24] Chang Y, Woo HG, Park J, Lee JS, Song TJ. Improved oral hygiene care is associated with decreased risk of occurrence for atrial fibrillation and heart failure: A nationwide population-based cohort study. Eur J Prev Cardiol. 2020;27(17):1835–45. 10.1177/2047487319886018. 31786965 10.1177/2047487319886018

[25] Kim J, Kim HJ, Jeon J, Song TJ. Association between oral health and cardiovascular outcomes in patients with hypertension: a nationwide cohort study. J Hypertens. 2022;40(2):374–81. 10.1097/HJH.0000000000003022. 34670996 10.1097/HJH.0000000000003022

[26] Huh Y, Yoo JE, Park SH, Han K, Kim SM, Park HS, et al. Association of dental diseases and oral hygiene care with the risk of heart failure in patients with type 2 diabetes: a nationwide cohort study. J Am Heart Assoc. 2023;12(16):e029207. 10.1161/JAHA.122.029207. 37548156 10.1161/JAHA.122.029207 PMC10492939

[27] Song TJ, Jeon J, Kim J. Cardiovascular risks of periodontitis and oral hygiene indicators in patients with diabetes mellitus. Diabetes Metab. 2021;47(6):101252. 10.1016/j.diabet.2021.101252. 33862198 10.1016/j.diabet.2021.101252

[28] Kobayashi D, Mizuno A, Mitsui R, Shimbo T. Frequency of daily tooth brushing and subsequent cardiovascular events. Coron Artery Dis. 2020;31(6):545–9. 10.1097/MCA.0000000000000882. 32310852 10.1097/MCA.0000000000000882

[29] Kajikawa M, Nakashima A, Maruhashi T, Iwamoto Y, Iwamoto A, Matsumoto T, et al. Poor oral health, that is, decreased frequency of tooth brushing, is associated with endothelial dysfunction. Circ J. 2014;78(4):950–4. 10.1253/circj.cj-13-1330. 24500034 10.1253/circj.cj-13-1330

[30] Del Pinto R, Pietropaoli D, Grassi G, Lorenza Muiesan M, Monaco A, Cossolo M, et al. Home oral hygiene is associated with blood pressure profiles: results of a nationwide survey in Italian pharmacies. J Clin Periodontol. 2022;49(12):1234–43. 10.1111/jcpe.13720. 36089901 10.1111/jcpe.13720 PMC9826426

[31] Wang Y, Jiang Y, Chen Y, Yu L, Zhou J, Wang N, et al. Associations of oral hygiene with incident hypertension and type 2 diabetes mellitus: a population based cohort study in Southwest China. J Clin Hypertens. 2022;24(4):483–92. 10.1111/jch.14451. 10.1111/jch.14451 PMC8989750 35255181

[32] Kuwabara M, Motoki Y, Ichiura K, Fujii M, Inomata C, Sato H, et al. Association between toothbrushing and risk factors for cardiovascular disease: a large-scale, cross-sectional Japanese study. BMJ Open. 2016;6(1):e009870. 10.1136/bmjopen-2015-009870. 26769787 10.1136/bmjopen-2015-009870 PMC4735199

[33] Tanaka A, Takeuchi K, Furuta M, Takeshita T, Suma S, Shinagawa T, et al. Relationship of toothbrushing to metabolic syndrome in middle-aged adults. J Clin Periodontol. 2018;45(5):538–47. 10.1111/jcpe.12876. 29421856 10.1111/jcpe.12876

[34] Kim YH, Kim DH, Lim KS, Ko BJ, Han BD, Nam GE, et al. Oral health behaviors and metabolic syndrome: the 2008–2010 Korean National health and nutrition examination survey. Clin Oral Investig. 2014;18(5):1517–24. 10.1007/s00784-013-1112-2. 24061606 10.1007/s00784-013-1112-2

[35] Tsutsumi C, Kakuma T. Regular tooth brushing is associated with a decreased risk of metabolic syndrome according to a medical check-up database. Kurume Med J. 2015;61(3–4):43–52. 10.2739/kurumemedj.MS64004. 25810422 10.2739/kurumemedj.MS64004

[36] Esfanjani MAT, Gilani N, Esfanjani AT, Nourizadeh AM, Faramarzi E, Hekmatfar S. Are oral health behaviors associated with metabolic syndrome in the Azar cohort population? BMC Oral Health. 2023;23(1):370. 10.1186/s12903-023-03003-0. 37291532 10.1186/s12903-023-03003-0 PMC10251534

[37] Chapple IL, Van der Weijden F, Doerfer C, Herrera D, Shapira L, Polak D, et al. Primary prevention of periodontitis: managing gingivitis. J Clin Periodontol. 2015;42(Suppl 16):S71–6. 10.1111/jcpe.12366. 25639826 10.1111/jcpe.12366

[38] Zimmermann H, Zimmermann N, Hagenfeld D, Veile A, Kim TS, Becher H. Is frequency of tooth brushing a risk factor for periodontitis? A systematic review and meta-analysis. Community Dent Oral Epidemiol. 2015;43(2):116–27. 10.1111/cdoe.12126. 25255820 10.1111/cdoe.12126

[39] Preshaw PM, Alba AL, Herrera D, Jepsen S, Konstantinidis A, Makrilakis K, Taylor R. Periodontitis and diabetes: a two-way relationship. Diabetologia. 2012;55(1):21–31. 10.1007/s00125-011-2342-y. 22057194 10.1007/s00125-011-2342-y PMC3228943

[40] Wang Z, Yang T, Fu H. Prevalence of diabetes and hypertension and their interaction effects on cardio-cerebrovascular diseases: a cross-sectional study. BMC Public Health. 2021;21(1):1224. 10.1186/s12889-021-11122-y. 34172039 10.1186/s12889-021-11122-y PMC8229421

[41] Passarella P, Kiseleva TA, Valeeva FV, Gosmanov AR. Hypertension management in diabetes: 2018 update. Diabetes Spectr. 2018;31(3):218–24. 10.2337/ds17-0085. 30140137 10.2337/ds17-0085 PMC6092891

[42] Barros AA, Guedes MVC, Moura DDJM, Menezes LCGD, Aguiar LL, Xavier GA. Health behaviors of people with hypertension: health belief model. Northeast Netw Nurs J. 2014;15(3):525–32. 10.15253/2175-6783.2014000300018.

[43] Genco RJ, Borgnakke WS. Risk factors for periodontal disease. Periodontol 2000. 2013;62(1):59–94. 10.1111/j.1600-0757.2012.00457.x. 23574464 10.1111/j.1600-0757.2012.00457.x

[44] Sheiham A, Watt RG. The common risk factor approach: a rational basis for promoting oral health. Community Dent Oral Epidemiol. 2000;28(6):399–406. 10.1034/j.1600-0528.2000.028006399.x. 10.1034/j.1600-0528.2000.028006399.x 11106011

