# Supplementary material for: Association of tooth brushing behavior with risks of major chronic health outcomes: a scoping review
Source: BMC Oral Health. 2025 Jun 8;25:943. doi: 10.1186/s12903-025-06332-4 (PMC12147352; doi:10.1186/s12903-025-06332-4)
Supplement: Supplementary file 1 — Supplementary Material 1 [file 12903_2025_6332_MOESM1_ESM.docx]

**Appendix. Search Terms**

| Ovid-Medline | Search strategy | No. |
| --- | --- | --- |
| 1 | exp Oral Hygiene/ | 21,166 |
| 2 | exp Dental Devices, Home Care/ | 2,089 |
| 3 | exp Toothbrushing/ | 8,332 |
| 4 | (toothbrush$ or tooth-brush$ or "tooth brush$").mp. | 11,045 |
| 5 | ((interdental adj3 brush$) or (interspace adj3 brush$) or (interproximal adj3 brush$)).mp. | 246 |
| 6 | ((interdental adj3 clean$) or (interspace adj3 clean$)).mp. | 268 |
| 7 | (interproximal adj3 clean$).mp. | 65 |
| 8 | or/ 1-7 | 23,243 |
| 9 | exp Chronic Disease/ | 316,799 |
| 10 | exp Heart Diseases/ | 1,288,049 |
| 11 | exp Coronary Disease/ | 238,658 |
| 12 | exp Cerebrovascular Disorders/ | 436,623 |
| 13 | exp Stroke/ | 178,430 |
| 14 | exp Diabetes Mellitus, Type 2/ | 177,060 |
| 15 | exp Metabolic Syndrome/ | 39,089 |
| 16 | exp Kidney Diseases/ | 582,064 |
| 17 | exp Renal Insufficiency, Chronic/ | 137,061 |
| 18 | or/ 9-17 | 3,760,475 |
| 19 | 8 and 18 | 1,265 |
| 20 | exp humans/ not animals.sh. | 19,525,788 |
| 21 | 19 and 20 | 1,295 |

| Embase | Search strategy | No. |
| --- | --- | --- |
| 1 | oral hygiene'/exp | 34,914 |
| 2 | Dental Devices, Home Care'/exp | 170,637 |
| 3 | Toothbrushing'/exp | 14,568 |
| 4 | (toothbrush*):ab,ti OR (tooth-brush*):ab,ti OR (tooth brush*):ab,ti | 10,102 |
| 5 | ((interdental NEAR/5 brush*):ab,ti) OR ((interspace NEAR/5 brush*):ab,ti) OR ((interproximal NEAR/5 brush*):ab,ti) | 380 |
| 6 | ((interdental NEAR/5 clean*):ab,ti) OR ((interspace NEAR/5clean*):ab,ti) | 338 |
| 7 | (interproximal NEAR/5 clean*):ab,ti | 78 |
| 8 | #1 OR #2 OR #3 OR #4 OR #5 OR #6 OR #7 | 209,217 |
| 9 | Chronic Disease'/exp | 255,147 |
| 10 | Heart Diseases'/exp | 2,546,132 |
| 11 | Coronary Disease'/exp | 438,870 |
| 12 | Cerebrovascular Disorders'/exp | 980,077 |
| 13 | Stroke'/exp | 469,795 |
| 14 | Diabetes Mellitus, Type 2'/exp | 373,707 |
| 15 | Metabolic Syndrome'/exp | 108,515 |
| 16 | Kidney Diseases'/exp | 1,339,832 |
| 17 | Renal Insufficiency, Chronic'/exp | 228,465 |
| 18 | #9 OR #10 OR #11 OR #12 OR #13 OR #14 OR #15 OR #16 OR #17 | 7,660,696 |
| 19 | #8 AND #18 | 9,779 |
| 20 | #19 AND 'human'/de AND 'article'/it AND [embase]/lim | 3,272 |

| Cochrane | Search strategy | No. |
| --- | --- | --- |
| 1 | MeSH descriptor: [Oral Hygiene] explode all trees | 2,483 |
| 2 | MeSH descriptor: [Dental Devices, Home Care] explode all trees | 401 |
| 3 | MeSH descriptor: [Toothbrushing] explode all trees | 1,544 |
| 4 | (toothbrush*):ab,ti OR (tooth-brush*):ab,ti OR (tooth brush*):ab,ti | 5,261 |
| 5 | ((interdental NEAR/5 brush*):ab,ti) OR ((interspace NEAR/5 brush*):ab,ti) OR ((interproximal NEAR/5 brush*):ab,ti) | 250 |
| 6 | ((interdental NEAR/5 clean*):ab,ti) OR ((interspace NEAR/5 clean*):ab,ti) | 191 |
| 7 | (interproximal NEAR/5 clean*):ab,ti | 43 |
| 8 | #1 or #2 or #3 or #4 or #5 or #6 or #7 | 6,454 |
| 9 | MeSH descriptor: [Chronic Disease] explode all trees | 17,399 |
| 10 | MeSH descriptor: [Heart Diseases] explode all trees | 62,024 |
| 11 | MeSH descriptor: [Coronary Disease] explode all trees | 15,654 |
| 12 | MeSH descriptor: [Cerebrovascular Disorders] explode all trees | 20,427 |
| 13 | MeSH descriptor: [Stroke] explode all trees | 14,140 |
| 14 | MeSH descriptor: [Diabetes Mellitus, Type 2] explode all trees | 22,959 |
| 15 | MeSH descriptor: [Metabolic Syndrome] explode all trees | 2,407 |
| 16 | MeSH descriptor: [Kidney Diseases] explode all trees | 19,772 |
| 17 | MeSH descriptor: [Renal Insufficiency, Chronic] explode all trees | 8,310 |
| 18 | #9 or #10 or #11 or #12 or #13 or #14 or #15 or #16 or #17 | 199,545 |
| 19 | #8 and #18 | 171 |

| CINAHL | Search strategy | No. |
| --- | --- | --- |
| 1 | AB ( (Oral Hygiene) or (Dental Devices, Home Care) or (Toothbrushing) ) AND AB ( (Chronic Disease) or  (Heart Diseases) or (Coronary Disease) or (Cerebrovascular Disorders) or (Stroke) or (Diabetes Mellitus, Type 2) or (Metabolic Syndrome) or (Kidney Diseases) or (Renal Insufficiency, Chronic)) | 385 |
